# Variations in the Minimum Joint Distance During Flexion and Abduction of the Thumb Carpometacarpal Joint: Potential Implications for Joint Degeneration

**DOI:** 10.7759/cureus.98044

**Published:** 2025-11-28

**Authors:** Masashi Matsuta, Mika Akahane, Akihiro Kurosawa, Kaoru Tada, Hiroshi Tachiya, Atsuro Murai, Yuta Nakamura, Hiroki Kawashima, Satoru Demura, Hiroyuki Tsuchiya

**Affiliations:** 1 Department of Orthopaedic Surgery, Graduate School of Medical Sciences, Kanazawa University, Kanazawa, JPN; 2 Faculty of Mechanical Engineering, Institute of Science and Engineering, Kanazawa University, Kanazawa, JPN; 3 Faculty of Health Sciences, Institute of Medical, Pharmaceutical and Health Sciences, Kanazawa University, Kanazawa, JPN

**Keywords:** biomechanics, carpometacarpal joint, joint distance, osteoarthritis, thumb

## Abstract

Background: The type of movement leading to osteoarthritis of the thumb carpometacarpal joint remains unclear. This study investigated changes in the joint distance during thumb flexion and abduction.

Methods: Five healthy adult men underwent computed tomography imaging at eight different limb positions. The joint distances perpendicular to the articular surfaces of the metacarpal (Dm) and trapezium (Dt) were measured.

Results: For Dm, the minimum joint distance was the smallest in both the flexion and abduction positions with narrowing predominantly observed on the volar side of the metacarpal. Similarly, for Dt, the minimum joint distance was also smallest in the flexion and abduction positions. Dm was mainly distributed on the volar side in all limb positions and tended to shift toward the radial side during abduction. Dt was generally located at the center and shifted toward the volar side in the flexion position.

Conclusion: Flexion and abduction of the thumb may contribute to degeneration of the volar side of the metacarpal articular surface. In particular, during maximum flexion, narrowing on the volar side of the trapezium was observed, indicating that flexion may contribute to degeneration not only in the central region but also on the volar side of the trapezium.

## Introduction

The carpometacarpal (CMC) joint of the thumb is a saddle-shaped joint capable of complex movements with a high degree of freedom. This joint enables thumb opposition, which is indispensable for grasping objects. Frequent use of the thumb places the CMC joint under considerable strain during daily activities, making it particularly susceptible to osteoarthritis. In the future, the number of patients with osteoarthritis of the CMC joint of the thumb is expected to increase with the aging population. However, the type of movement that causes osteoarthritis of the CMC joint of the thumb has not been clarified. Therefore, elucidating the mechanisms underlying the onset and progression of osteoarthritis of the thumb CMC joint may allow the development of preventive and effective treatments. Osteoarthritis is thought to begin with cartilage wear, and one of the causes of this cartilage wear is thought to be that the distance between joints becomes narrower and the load on the cartilage increases. Alternatively stated, it can be said that movements that narrow the joint distance contribute to the development of osteoarthritis. Eaton et al. [1,2] reported that the minimum joint distance of the joints that led to osteoarthritis was close to zero. However, previous imaging studies have largely examined only the final positions of motion and the dynamic changes in minimum joint distance during the process of thumb motion remain unclear [3,4]. Consequently, it is not yet known which specific thumb positions generate the greatest compressive conditions at the metacarpal and trapezial articular surfaces.

To address this knowledge gap, our study aimed to quantitatively characterize the minimum joint distance of the thumb CMC joint across multiple intermediate positions during both extension-flexion and adduction-abduction movements. By clarifying the joint positions that generate the greatest narrowing, this study provides biomechanical insight into movement patterns that may contribute to the onset or progression of CMC joint osteoarthritis.

## Materials and methods

**Figure 1:**
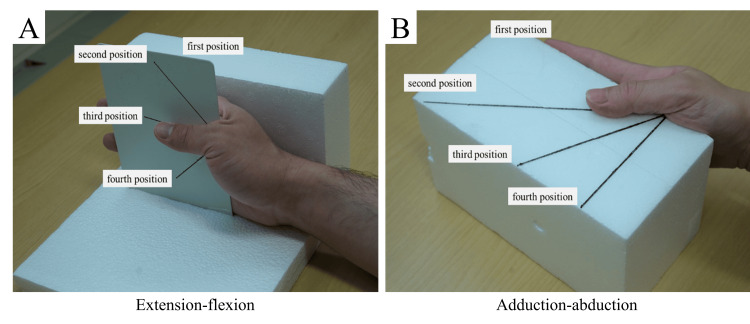
Photograph of the self-made assistive device used for CT imaging of thumb flexion and abduction. This device enables vertical and horizontal movement with respect to the palm. A shows the device for extension-flexion, and B shows the device for adduction-abduction. This figure was created by the authors.

**Figure 2:**
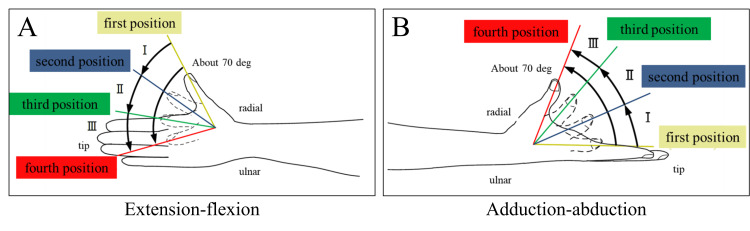
Definition of thumb flexion and abduction positions. The flexion and abduction movements of the thumb were divided into three equal parts, and each limb position was defined as shown. A shows extension-flexion movement, and B shows adduction-abduction movement. This figure was created by the authors.

**Figure 3:**
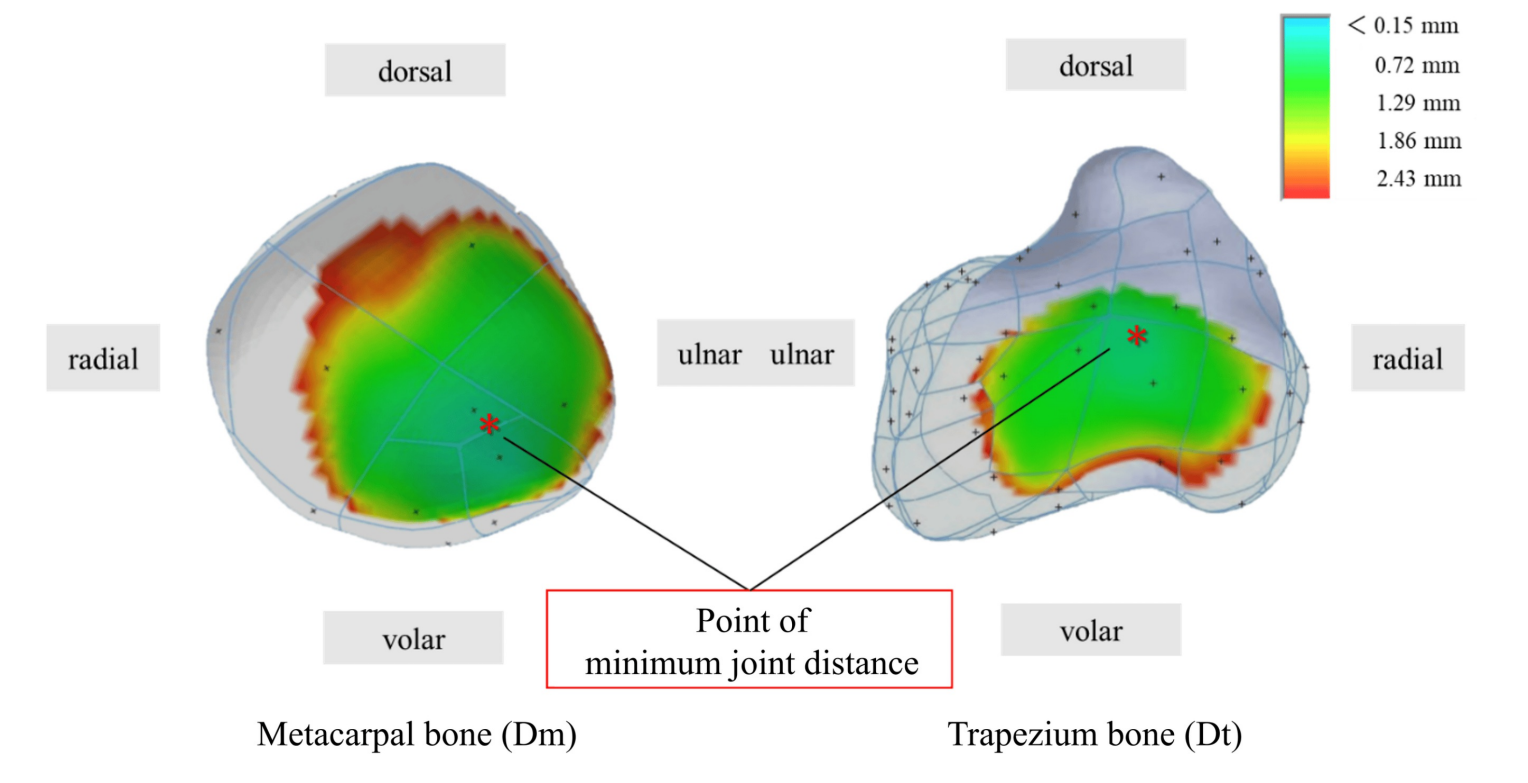
Joint distance color mapping on the articular surfaces. The joint distances Dm and Dt were mapped to the articular surfaces of the metacarpal and trapezium bones, respectively, based on a custom color scale. This figure was created by the authors.

**Figure 4:**
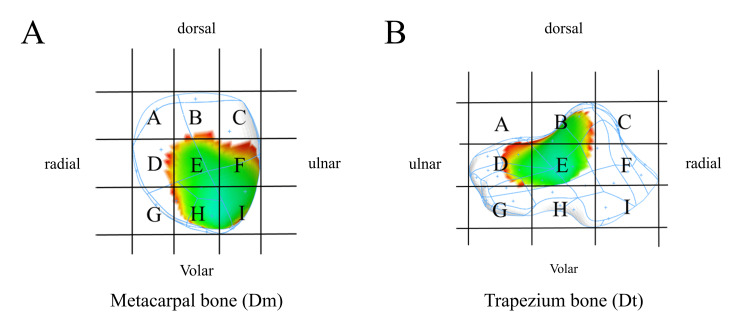
Nine-region division of the articular surfaces. The articular surfaces were divided into nine regions by trisecting the dorsal and radial sides. In A, each divided region of the metacarpal articular surface is labeled with the letters A to I, and in B, each divided region of the trapezium articular surface is labeled with the letters A to I. This figure was created by the authors.

This study was approved by the ethics committee of our institution (approval number: 2621). Informed consent was obtained from all the participants included in this study. This study was conducted as a pilot investigation to evaluate the technical feasibility of minimum joint distance in multiple thumb positions. The participants were five healthy adult men involving five right hands from participants with a mean age of 44.4 (standard deviation, 14.9) years (Table 1). All participants were white-collar workers who primarily performed desk-based tasks. Exclusion criteria for this study included several comorbidities such as hand or thumb surgery, traumatic injury, inflammatory arthritis, metabolic bone disease, and connective tissue disease. Computed tomography (CT) imaging of the four limb positions during flexion and abduction of the thumb was performed at 180° of shoulder flexion, 0° of elbow extension and the middle position in pronation and supination of the forearm, using a self-made assistive device to stabilize the thumb position during imaging (Figure 1). This device, which was developed specifically for this study, was intended solely to enhance posture reproducibility during CT imaging. The movement from the maximum extension position to the maximum flexion position of the thumb was divided into three equal parts: the maximum extension position is the first position, the maximum flexion position is the fourth position, and the intermediate limb position is the second and third positions. Similarly, the movement from the maximum adduction position to the maximum abduction position of the thumb was divided into three equal parts: the maximum internal displacement is the first position, the maximum external displacement is the fourth position, and the intermediate limb position is the second and third positions (Figure 2). A 128-slice multidetector CT (SOMATOM Definition Flash, Siemens Healthineers) was used for CT imaging, and the imaging conditions were 2.0 seconds per limb position, 0.75 mm slice thickness, 0.3 mm slice interval, 20 mAs, 100 kV tube voltage, and 0.822 mGy as the Computed Tomography Dose Index. The effective dose associated with CT imaging was approximately 0.004 mSv per limb position. The CT scanner’s standard factory calibration was used. A three-dimensional (3D) model of the CMC joint of the thumb was created based on the obtained CT images. All segmentations and 3D reconstructions were performed by a single trained operator to ensure consistency. The minimum joint distance was calculated using a 3D surface point projection method. For each articular surface, a point-to-surface distance search was performed in which the distance from every surface point on one bone to the opposing articular surface was computed. The joint distance Dm is defined as the distance measured perpendicular to the articular surface of the metacarpal bone, while the joint distance Dt is defined as the distance measured perpendicular to the articular surface of the trapezium. We mapped them to the articular surface based on a custom color scale. (Figure 3). The thickness scale was 0.15 mm to 3.00 mm. 3D CAD software was used as the analysis software. The coordinate axes were set using standardized joint coordinate systems (JCS) by the International Society of Biomechanics (ISB) [5], and the minimum joint distance and its distribution in each limb position were evaluated. The distribution of the minimum joint distance was based on anatomical landmarks on the proximal side of the JCS for the metacarpal bones and the distal side of the JCS for the trapezium. The distribution of the minimum of the joint distance was evaluated by nine regions divided into three equal parts on the dorsal and the radial sides (Figure 4). IBM SPSS Statistics for Windows, Version 24 (Released 2016; IBM Corp., Armonk, New York, United States) was used as the statistical software, and Dunnett's test was performed with the target category as the fourth position, which is the final limb position of each movement. A significant difference was observed at P <0.05.

**Table 1:** Demographic characteristics of the participants. This table shows the sex, age, and examined side of the participants.

Case number	Sex	Age	Examined side
No. 1	Male	24 years	Right
No. 2	Male	31 years	Right
No. 3	Male	50 years	Right
No. 4	Male	52 years	Right
No. 5	Male	65 years	Right

## Results

**Figure 5:**
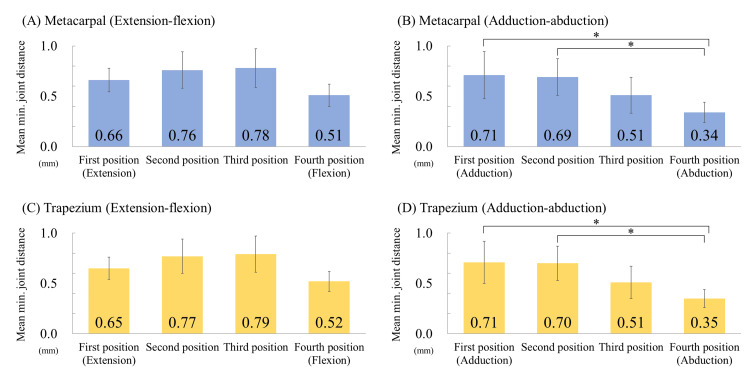
Graph of the mean minimum joint distance at each limb position. The mean minimum joint distance was calculated for each limb position during thumb flexion and abduction. A and B represent distances measured perpendicular to the articular surface of the metacarpal bone, whereas C and D represent distances measured perpendicular to the articular surface of the trapezium. Error bars indicate standard deviations, and * denotes P < 0.05.

The mean minimum joint distance of Dm during extension-flexion movement was 0.66 mm in the first position, 0.76 mm in the second, 0.78 mm in the third, and 0.51 mm in the fourth (Figure 5A). The mean minimum joint distance of Dm during adduction-abduction movement was 0.71 mm in the first position, 0.69 mm in the second, 0.51 mm in the third, and 0.34 mm in the fourth (Figure 5B). The mean minimum joint distance of Dt during extension-flexion movement was 0.65 mm in the first position, 0.77 mm in the second, 0.79 mm in the third, and 0.52 mm in the fourth (Figure 5C). The values increased when the thumb moved from extension to flexion and then decreased at the maximum flexion position. However, no significant difference was observed between the limb positions. The mean minimum joint distance of Dt during adduction-abduction movement was 0.71 mm in the first position, 0.70 mm in the second, 0.51 mm in the third, and 0.35 mm in the fourth (Figure 5D). The values gradually decreased from adduction to abduction and were significantly smaller in the fourth position than in the first and second.

The distributions of the minimum joint distance of Dm were often present on the volar side of the metacarpal bone in all limb positions; especially in the abduction position, the distributions tended to shift toward the radial side (Table 2, Table 3). The distributions of the minimum joint distance of Dt were often present in the center of the trapezium bone in all limb positions. In the flexion position, distributions tended to shift toward the volar side compared with extension (Table 4, Table 5).

**Table 2:** The results of the distribution of the minimum of the joint distance on the metacarpal articular surface during extension–flexion movement. The letters in the table represent the regions defined by Figure 4.

Metacarpal (Extension-flexion)				
	First position (Extension)	Second position	Third position	Fourth position (Flexion)
No. 1	G (border with H)	G	G	G
No. 2	I	I (border with H)	H	H
No. 3	F	F	H	G (border with H)
No. 4	H	H	H	H
No. 5	E	I	I	H

**Table 3:** The results of the distribution of the minimum of the joint distance on the metacarpal articular surface during adduction-abduction movement. The letters in the table represent the regions defined by Figure 4.

Metacarpal (Adduction-abduction)				
	First position (Adduction)	Second position	Third position	Fourth position (Abduction)
No. 1	H	H	G	D
No. 2	I	H (border with G)	G	D
No. 3	E (border with B)	E (border with D)	D	D
No. 4	H (border with I)	H	D (border with G, H)	H (border with D)

**Table 4:** The results of the distribution of the minimum of the joint distance on the trapezium articular surface during extension–flexion movement. The letters in the table represent the regions defined by Figure 4.

Trapezium (Extension-flexion)				
	First position (Extension)	Second position	Third position	Fourth position (Flexion)
No. 1	B	E	E	H
No. 2	D	D (border with E)	H	H
No. 3	D	D	G (border with H)	H
No. 4	E	E	E	H
No. 5	B	B	E	G (border with D)

**Table 5:** The results of the distribution of the minimum of the joint distance on the trapezium articular surface during adduction-abduction movement. The letters in the table represent the regions defined by Figure 4.

Trapezium (Adduction-abduction)				
	First position (Adduction)	Second position	Third position	Fourth position (Abduction)
No. 1	E	E	H	E
No. 2	E	E	H	E
No. 3	B	E	E	E
No. 4	E	E	E	E
No. 5	E	D	E	E

## Discussion

Few studies have investigated changes in joint distance across multiple limb positions. D'Agostino et al. [3] evaluated the metacarpal joint space, which is the joint distance perpendicular to the metacarpal joint surface of the thumb in four limb positions: maximal extension, maximal flexion, maximal abduction, and maximal adduction. The joint distance of the metacarpal bone was larger in the adduction and extension positions than in the abduction and flexion positions, and the results were similar to those in this study. Halilaj et al. [4] discussed the distance between the metacarpal joint and the trapezium joint spaces of the CMC joint, which is the joint distance perpendicular to the trapezium joint surface of the thumb in the four positions of extension, flexion, abduction, and adduction using their orthoses. The joint distance of the metacarpal bones tended to be smaller on the volar side in all limb positions. The joint distance of the trapezium bone was small on the dorsal side in the extension position, and was small on the volar side in the flexion, abduction, and adduction positions, and the position in the radial direction did not differ depending on the limb position. In these studies on the joint distance, many images were taken in the final limb position of the movement, and it was not clarified how the joint distance changes in the process of extension-flexion movement and adduction-abduction movement. The processes of extension-flexion movement and adduction-abduction movement were taken in four positions divided into three parts in this study. As a result, both the minimum joint distances, Dm and Dt, exhibited a slight increase from the extension to the intermediate position during extension-flexion movement, reached their minimum values at maximum flexion, and progressively decreased from adduction to abduction during adduction-abduction movement.

In this study, flexion and abduction movements tended to narrow the volar side of the metacarpal joint surface. Although our imaging conditions do not replicate functional loading, these positional patterns may be associated with regions known to exhibit cartilage wear in clinical cases. Thus, deep flexion and deep abduction may represent mechanical environments that contribute to increased compressive forces, potentially influencing degenerative changes over time. Importantly, these findings should be interpreted as associations rather than direct causal mechanisms, given the static and non-weight-bearing nature of our imaging. Multiple clinical and biomechanical studies have described degeneration patterns on the volar side of the metacarpal bone. Pellegrini [6] reported that the flexion-adduction of the thumb in the lateral pinch causes the metacarpal bones of the thumb to shift to the dorsal side, to increase joint laxity, and to generate shearing force in the contact area on the volar side, leading to degeneration of the palmar ligament. Goto et al. [7] showed that the limb position of the thumb tends to narrow in the center of the volar side due to key pinch, palmar abduction, and opposition. Edmunds [8] reported that in the CMC joint of the thumb, stable thumb opposition is possible by engaging the beak of the metacarpal bone and the recess of the trapezium. Degeneration of the palmar beak ligament was previously thought to be the cause of osteoarthritis of the CMC joint. However, Edmunds [8] stated that the palmar beak ligament is thin, weak, and relaxed during the opposition position, while the dorsal ligament complex is thick, strong, and serves as an important stabilizing ligament that is tensed during opposition for power grip and power pinch. Therefore, damage to the dorsal ligament complex, due to trauma or similar factors, can lead to instability of the CMC joint even if the palmar beak ligament remains intact. Tada et al. [9] and D'Agostino et al. [10] described the screw-home torque rotation, a characteristic movement of the CMC joint of the thumb. They reported that during extension-flexion movement, the metacarpal flexes and rotates inward at maximum flexion, thereby increasing joint congruency. This congruency generates compressive forces on the volar articular surface, which is believed to contribute to the development of osteoarthritis of the CMC joint of the thumb. This screw-home torque rotation corresponds to the movement in which the joint distance was most reduced in our study, and therefore, maximal flexion and maximal abduction are considered the primary movements responsible for CMC joint degeneration.

There are various views on where the trapezium bone is prone to degeneration. Lee et al. [11] evaluated the trabecula of the trapezium in patients with osteoarthritis of the CMC joint of the thumb by micro-CT and inferred that the pressure on the volar side increased and it was prone to degeneration. Ateshian et al. [12] evaluated the thickness of the cartilage and speculated that the volar, ulnar, and dorsal sides of the trapezium were thin and prone to degeneration. Xu et al. [13] stated that the dorsal side is less likely to degenerate morphologically. In contrast, Kovler et al. [14] investigated cartilage damage using an anatomical microscope and reported that cartilage damage was strong on the dorsal side of the trapezium bone. Van Nortwick et al. [15] reported that the trapezium wear patterns were saddle-shaped, dish-shaped, and cirque-shaped. In this study, the minimum joint distance Dt tended to decrease in the maximum flexion and abduction positions. It was distributed in the central region during abduction and on the volar side during flexion. In some cases, Dt was distributed on the ulnar side during extension, but none of the cases showed distribution on the radial side. These findings suggest that repeated flexion movements may promote degeneration not only in the central region but also on the volar side. Compared with previous reports, the results of this study are in agreement with the dish-shaped wear pattern reported by Van Nortwick et al. [15] that the ulnar side is easily degenerated and the radial side is not. This is partly in agreement with the findings of Lee et al. [11] and Ateshian et al. [12].

The first limitation of this study is the small sample size, as it was designed as a feasibility-oriented pilot investigation rather than a powered analysis. Although inter-rater reliability was not assessed, all semi-automated segmentations were performed by a single operator to ensure consistency. Despite the small cohort, the observed patterns, minimum joint distance on the volar metacarpal side and central trapezium, were consistent across all participants. Nevertheless, the small, all-male sample limits the generalizability of our findings. Therefore, future studies with larger cohorts will be necessary to confirm these findings and address variations reported in previous literature. Second, the participants in this study were limited to men. However, Halilaj et al. [16,17] reported that there was no age or gender difference in morphology or instability, and Crisco et al. [18] reported that there was no difference in kinematic variables between genders. However, the absence of female participants still restricts reproducibility and may overlook subtle gender-specific biomechanical variations. We acknowledge that including women in future studies will be crucial for improving both external validity and applicability to the broader clinical population. Third, in this study, measurements were performed using CT images, and it is a measurement of the subchondral bone. Although magnetic resonance imaging (MRI) could theoretically allow for direct cartilage assessment, its spatial resolution and acquisition time pose challenges for small joints such as the thumb CMC joint. Integrating advanced imaging techniques, including high-resolution MRI, may help overcome this limitation in future research. Fourth, because it is a static uniaxial movement, it is not based on activities of daily living, and the distance between joints when force is applied cannot be evaluated. Osteoarthritis of the CMC joint of the thumb is caused by repetitive thumb movements in daily life. This result alone is not necessarily the cause of osteoarthritis of the CMC joint. In the future, it will be necessary to evaluate the distance during pinch and grip movements, and not only the distance between joints but also the pressure applied between joints in the eight limb positions and examine whether similar results can be obtained.

## Conclusions

This study demonstrated that the thumb CMC joint exhibited its minimum joint distances at maximal flexion and abduction, with narrowing particularly on the volar side of the metacarpal articular surface. During maximum flexion, narrowing was also observed on the volar side of the trapezium. These positional changes may be associated with mechanical environments that impose increased compressive forces on these particular regions. the observed narrowing patterns provide insight into joint positions that may contribute to biomechanical conditions relevant to the development and progression of thumb CMC osteoarthritis.
